# Is ‘too much medicine’ a guideline‐driven phenomenon? Ten years' report and reflections of the Guidelines International Network Multimorbidity Working Group

**DOI:** 10.1002/gin2.12016

**Published:** 2024-06-03

**Authors:** Martin Scherer, Jako S. Burgers, the Guidelines International Network Multimorbidity Working Group

**Affiliations:** ^1^ Department of Primary Medical Care University Medical Center Hamburg Eppendorf Germany; ^2^ Department of Community Health and Family Medicine University of Florida Gainesville Florida USA; ^3^ Dutch College of General Practitioners Utrecht The Netherlands; ^4^ Department of Family Medicine, Care and Public Health Research Institute Maastricht University Maastricht The Netherlands

**Keywords:** climate change, clinical practice guidelines, disease burden, multimorbidity, polypharmacy

## Abstract

- The Guidelines International Network Multimorbidity Working Group aims to explore how and to what extent multimorbidity can be reflected in guideline development.
- Single disease guidelines contribute to ‘too much medicine’, including the abundance of diagnostic and therapeutic measures with potential harm to the patient.
- The ‘less is more’ approach includes communication on patient outcomes, recommendations for deprescribing and awareness of climate impact.
- Guidelines supporting person‐centred care could reduce the risk of low‐value care for multimorbid patients, which will benefit both individual and planetary health.

## 1 INTRODUCTION

Established at the Guidelines International Network (GIN) annual conference in 2013 in San Francisco, the GIN Multimorbidity Working Group has existed for 10 years. Its aim was and is to explore the questions of how and to what extent multimorbidity and its related constructs can be reflected in guideline development. A major methodological challenge is to find a balance between not getting lost in the thousands of different disease combinations and formulating recommendations that are useful in everyday clinical practice.

## 2 HISTORY

Cynthia Boyd, founding member and first chair of the GIN multimorbidity working group, published a frequently cited article on the accumulation of guideline recommendations for an individual 72‐year‐old patient with multimorbidity. She described the complexity of the patient's condition and the number of diagnoses that can affect the number of guideline recommendations and thus maximize the treatment burden for those involved.^1^ Today, 18 years later, the topic of multimorbidity, with its associated phenomena such as polypharmacy, is well established and a topic of several guidelines.^2^, ^3^, ^4^, ^5^

## 3 DEFINITION

There are a variety of definitions of multimorbidity, from the simple counting of diagnoses to the use of complex indices that account for disease severity and drug therapy, biopsychosocial and somatic risk factors.^6^, ^7^ They all have in common that multimorbidity is defined as the simultaneous presence of several chronic diseases, with no one disease initially taking priority.^8^ In contrast, if one disease is dominant (called index disease), the term co‐morbidity is often used.^9^

## 4 MULTIMEDICINE

In recent years, it has been recognized that ‘too much medicine’ is being practiced overall and that many diagnostic and therapeutic measures are either superfluous at best or often even harmful.^10^ Since too much medicine is a widespread problem across all medical disciplines, it is obvious that the overwhelming number of symptoms and problems associated with multimorbidity can lead to an abundance of diagnostic and therapeutic measures. Moreover, too much medicine means an increase in treatment burden and potential harm for the multimorbid patient.

A key issue for the GIN Multimorbidity Working Group is how guidelines for multimorbidity can address this problem. How can we ensure that the necessary is done and the unnecessary avoided for people with multimorbidity? Last but not least, how can the ‘less‐is‐more approach’ to multimorbidity be implemented?

## 5 RESULTS OF THE GIN WORKING GROUP MEETING

At the GIN Annual Congress 2023 in Glasgow, the working group discussed the scope and purpose of guidelines, healthcare system issues and guideline developers' challenges.

### 5.1 Scope and purpose

The participants agreed that ‘less is more’ as well as the avoidance of overuse is an important topic for multimorbidity guidelines. There was also broad consensus that the prevention of overdiagnosis and overtreatment should be prioritized at a very early stage of multimorbidity management, such as the strict indication of diagnostic procedures and anticipation of the polypharmacy career before prescribing a medication. It was also pointed out that greater attention should be paid to identifying the risks of polypharmacy. The doctor–patient communication on the importance of outcomes should primarily follow the principles of patient‐centeredness and shared decision‐making. The benefits of deprescribing should be clearly explained in the guideline, both in terms of individual, societal and planetary health aspects. Deprescribing not only reduces the individual treatment burden but also positively affects carbon dioxide emissions in the long term, to which the healthcare sector contributes significantly.^11^ Data from the healthcare system in the United Kingdom (National Health Service) show that emissions from supply chains account for 62% of emissions. Most of these emissions are caused by medicines, medical equipment and consumables. The transportation of patients, staff and visitors also accounts for around 10% of emissions.^12^ Climate change is a global health issue.^13^, ^14^

### 5.2 Healthcare system

Guidelines as tools for knowledge translation and healthcare improvement should be seen within the context of the country's healthcare system. It was emphasized that service providers in the healthcare system often find themselves in a hamster wheel, which means providing care when the workload and number of patients are too high and the time is too short. This is intensified to a considerable extent in the case of multimorbidity. The potential of digital solutions to contribute to sustainable health was mentioned. Policymakers and payers in healthcare may assume that digital care will increase efficiency, but patients with multimorbidity need person‐centred and coordinated care.^15^ In addition, there was a warning about the risk that the rapidly growing and often profit‐oriented telemedicine market could even exacerbate the problem of overintervention.

### 5.3 Challenges

The working group was critical that the methodological challenges and tasks for guideline developers, which have been increasing for years, have led to an overscoping of the guidelines and thus to a serious side effect of guidelines, the reinforcement of ‘too much medicine’. It was recommended that the promotion of high‐value care should be a cross‐cutting task of all guidelines and at the same time, the risks for low‐value care should be worked out across all indications. After all, this is where co‐benefits for individual and planetary health lie.

## 6 DISCUSSION

The working group cited the hamster wheel phenomenon as a major system‐related flaw. Across healthcare systems and countries, every medical discipline suffers from a seemingly never‐ending workload. There is a danger of not being able to give patients the time and attention that they need, particularly patients with multimorbidity and complex problems. Guidelines contribute to this considerably.^16^, ^17^ A simulation study applying Grade A and B recommendations of the guidelines of the US Preventive Service Taskforce guidelines for preventive care, chronic disease care and acute care to a representative group of 2500 adults in the United States, estimated that primary care physicians would need up to 27 h per working day to implement all applicable guidelines.^18^ Several studies included in their review suggest that short consultation length was responsible for driving polypharmacy, overuse of antibiotics and poor communication with patients.^19^, ^20^, ^21^ Therefore, the ‘time needed to treat’ should be considered when making recommendations. Particularly in the case of multimorbidity, the time for the patient is not needed for the linear processing of guideline recommendations but for joint prioritization of complex problems to create a shared decision‐based care plan.^22^

Almost 10 years ago, the length of a guideline was considered in inverse proportion to its evidence‐based nature; in other words, the longer, the lower the value of care.^23^ Today, the picture on the guideline horizon is by no means better: the trend is towards more voluminous guidelines. For example, clinical practice guidelines in oncology have increased dramatically in quantity, complexity and growth over the past 2 decades. Between 1996 and 2019, the mean page count of the National Comprehensive Cancer Network Guidelines increased from 26 to 198 pages, a 762% absolute increase overall.^24^

There are a number of instruments that can be used to measure the methodological quality of guidelines.^25^, ^26^ The logic is that if specific methodological criteria are adhered to, the quality of the content of a guideline will automatically follow as a matter of course. This automatism cannot be relied upon.^27^ It is, therefore, urgently necessary to systematically evaluate the quality of the content of guidelines by addressing the high‐value care component.

Guidelines on multimorbidity could contain thousands of pages if all potential combinations of conditions were addressed. Therefore, it is paramount to limit the scope of the guideline and focus on the priority problems.^28^ Alternatively, one could formulate basic principles of good healthcare, emphasizing person‐centred, integrated care,^29^ shared decision‐making^30^ and coordination of care. Both studies and guidelines should generate more evidence for everyday practice that accounts for the complexity of multiple interrelated aspects of multimorbidity management.^31^ In addition, patients with significant treatment burden, which can result in poor adherence to treatment and adverse outcomes, should be identified and targeted. Less can be more, even in clinical practice guidelines.

## 7 CONCLUSION

After 10 years, the focus of the GIN Multimorbidity Group evolved from raising awareness on multimorbidity to finding sustainable solutions for guideline developers. Single disease guidelines contribute to too much medicine. Guidelines supporting person‐centred care might be considered as a solution if recommendations are flexible and facilitate shared decision‐making in clinical practice without increasing the treatment burden. Although multimorbid patients are unique, guidelines could offer better roadmaps to high‐value care.

## AUTHOR CONTRIBUTIONS

**Martin Scherer**: Conceptualization (lead); writing—original draft (lead); writing—review and editing (equal). **Jako S. Burgers**: Conceptualization (supporting); writing—original draft (supporting); writing—review and editing (equal). **GIN Multimorbidity Working Group**: Conceptualization (supporting); writing—review and editing (equal).

## CONFLICT OF INTEREST STATEMENT

The authors declare no conflict of interest.

## ETHICS STATEMENT

No ethical approval was needed for this study.

## Supporting information

Supporting information.

Supporting information.

## Data Availability

Data sharing is not applicable to this article as no data sets were generated or analysed during the current study.
